# Oxygen-Carbon Nanotubes as a Chemotherapy Sensitizer for Paclitaxel in Breast Cancer Treatment

**DOI:** 10.1371/journal.pone.0104209

**Published:** 2014-08-04

**Authors:** Yongkun Wang, Chuanying Wang, Yijun Jia, Xianhua Cheng, Qing Lin, Mingjie Zhu, Yunshu Lu, Longlong Ding, Ziyi Weng, Kejin Wu

**Affiliations:** 1 Department of General Surgery, XinHua Hospital, Shanghai Jiaotong University School of Medicine, Shanghai, China; 2 School of Mechanical and Power Engineering, Shanghai Jiaotong University, Shanghai, China; 3 Department of Oncology, XinHua Hospital, Shanghai Jiaotong University School of Medicine, Shanghai, China; 4 Department of Pathology, XinHua Hospital, Shanghai Jiaotong University School of Medicine, Shanghai, China; 5 Department of Anorectal Surgery, XinHua Hospital, Shanghai Jiaotong University School of Medicine, Shanghai, China; University of Nebraska Medical Center, United States of America

## Abstract

**Objective:**

To study the in vivo and in vitro effects of adding oxygen carbon nanotubes (CNTs) to chemotherapy for breast cancer.

**Methods:**

MCF-7 and SK-BR-3 breast cancer cells were co-cultured with paclitaxel and then exposed to oxygen-CNTs under hypoxic conditions. Cell proliferation, viability, and apoptosis rate were analyzed. Hypoxia-inducible factor-1 alpha (HIF-1α) expression was measured using reverse transcription-polymerase chain reaction (RT-PCR) and western blot. Nude mice were used as a human breast cancer model to explore the impact of oxygen-CNTs on the in vivo chemotherapeutic effect of paclitaxel.

**Results:**

Oxygen-CNTs had no significant effects on the growth of breast cancer cells under normoxia and hypoxia. However, in the hypoxic environment, oxygen-CNTs significantly enhanced the inhibitory effect of paclitaxel on cell proliferation, as well as the apoptosis rate. Under hypoxia, downregulation of HIF-1α and upregulation of caspase-3, caspase-8, caspase-9, LC3 and Beclin-1 were observed when paclitaxel was combined with oxygen-CNT. Furthermore, addition of oxygen-CNTs to chemotherapy was found to significantly reduce tumor weight in the tumor-bearing mice model.

**Conclusions:**

Oxygen-CNTs can significantly increase the chemotherapeutic effect of paclitaxel on breast cancer cells. Oxygen-CNTs may be a potential chemosensitizer in breast cancer therapy.

## Introduction

Breast cancer is one of the most common cancers in women, affecting 1.2 million women in the world and being responsible for the death of 500,000 women each year. China is among the countries with the fastest growing incidence of breast cancer, and statistical data from the Chinese Anti-Cancer Association show that in recent years, the incidence of breast cancer has been increasing by 3% every year, and has been accompanied by a decrease in the age of onset [1].

Despite great advances in breast cancer treatment, there are still cases of tumor resistance to treatments. Hypoxia is an important factor that affects the efficacy of treatment. Indeed, hypoxia significantly increases tumor resistance to chemotherapy and radiotherapy, and may also increase the invasiveness of the tumor [2], [3]. Hypoxia-inducible factor 1 alpha (HIF-1α) plays a key role in the effects of hypoxia in cancer cells. Indeed, HIF-1α expression is associated with survival of breast cancer patients after surgery [4]. Therefore, overcoming the effects of hypoxia might improve the efficacy of breast cancer treatments.

In recent years, nanotechnology has been used for a wide range of applications in medicine and biology. Carbon nanotubes (CNTs) are expected to achieve a breakthrough in the treatment of breast cancer and other tumors by reducing the tumors' resistance to radiotherapy and chemotherapy [5]–[7]. Functional CNTs have a potential for protein and gene transfer across the cell membrane and into the nucleus [8]–[17]. The present study is based on CNTs' characteristics that were developed using a mixed acid purification method. CNTs' surface is rich in hydroxyl and carboxyl functional groups, which increases the oxygen-binding capacity of these CNTs, making them efficient oxygen carriers.

We undertook the present study to test the hypothesis that oxygen-CNTs inhibit breast cancer cell proliferation induced by HIF1α. Our findings show that oxygen-CNTs are potent chemosensitizers that improve the effects of paclitaxel in vivo and in vitro. Our results further indicate that oxygen-CNTs induce this effect by improving the hypoxia status and sensitivity to chemotherapy.

## Materials and Methods

### Materials

Functional oxygen single-walled carbon nanotubes (SWCNTs) were provided by the Mechanical and Power Engineering College of Shanghai Jiaotong University (Shanghai, China). DMEM, RPMI 1640 medium, fetal bovine serum (FBS), penicillin and streptomycin were from GIBCO (Invitrogen Inc., Carlsbad, CA, USA).

### Ethical considerations

Nude mice were used as an animal model to study the biological effects of oxygen-CNTs. This study was carried out in strict accordance with the recommendations of the Guide for the Care and Use of Laboratory Animals of the National Institutes of Health. The protocol was approved by the Committee on the Ethics of Animal Experiments of the Xinhua Hospital, affiliated to Shanghai Jiaotong University School of Medicine, China. Surgeries were performed under sodium pentobarbital anesthesia, and all efforts were made to minimize suffering.

### CNT pretreatment, hydroxylation and X-ray photoelectron spectroscopy analysis

CNTs were purified by acid treatment and high energy ball mill; they were of >95% purity, 0.5–2 µm in length, and 10–20 nm in diameter. Single-wall carbon nanotubes (1 g) (XFNANO Materials Tech. Co., Ltd., Nanjing, China) were ground in an agate mortar for 1 h. Then, 0.1 g of the ground CNT powder was placed in a 500-mL Erlenmeyer containing 50 mL of an acid mixture (3∶1 v/v ratio of 98% sulfuric acid and 78% nitric acid), for a concentration of 2 mg of nanotubes per 1 ml of acid solution. The solution was treated in an oscillating ultrasonic bath (SKYMEN Cleaning Equipment Co., Ltd., Shenzhen, China) for 2 h at room temperature. Oxidative reflux was conducted for 0.5 h at 100°C, and then the reaction liquid were naturally cooled to room temperature, after which 300 mL of deionized water was added. The flask was kept still for some time until the precipitate containing oxygen-CNTs had deposited at the bottom of the flask. The supernatant was discarded, and the turbid liquid that remained was filtered through a millipore filter. The filter paper containing the oxygen-CNT residue was placed in a beaker containing deionized water, and was ultrasonicated (SKYMEN Cleaning Equipment Co., Ltd., Shenzhen, China) to separate the CNTs. The solution was poured into an Erlenmeyer and repeatedly washed, filtered and resuspended until the pH value was neutral. The oxygen-CNT-containing solution was then dried in an oven at 60°C. We obtained a black powder that was used for the further experiments.

### Nanotubes analysis

The constituent elements (oxygen, carbon and nitrogen) of the oxygen CNTs were detected by X-ray photoelectron spectroscopy (XPS) analysis.

Carbon nanotubes were dispersed, dropped on a copper screen and observed under a JEM-2010 high-resolution transmission electron microscope (TEM) to assess their distribution in solution.

### Cell culture

The breast cancer cell lines MCF-7 and SK-BR-3 were purchased from the Shanghai Cell Bank of the Chinese Academy of Sciences (Shanghai, China). Cells were cultured in DMEM and RPMI1640 complete media (containing 10% FBS, 100 U/L of penicillin, and 100 U/L of streptomycin). The cultures were incubated in a normoxic incubator (37°C, 5% CO_2_) for 12h and then in a hypoxic incubator (37°C, 5% CO_2_, 1% O_2_, 94% N_2_) for 48 h. Adherent cells were picked up from the culture and subjected to 0.25% trypsin digestion. Cells in the logarithmic growth phase were used for subsequent experiments.

### Cell proliferation

The water-soluble tetrazolium salt assay (WST-1; Nanjing KeyGen Biotech Co., Nanjing, China) was used to determine cell viability. To conduct the assay, 10 µL of WST-1 (5 mg/mL) working solution was added to each well at a final concentration of 500 µg/mL. After the mixture in each well was incubated for 2.5 h, the absorbance was read with a microplate reader (uQuant; Bio-Tek Services Inc., Richmond, VA, USA) at 450 nm. Relative cell viability was expressed as the percentage of viable cells divided by the number of viable cells in the control wells that were not treated with functional CNTs. The inhibition rate was calculated using the formula: cell inhibition rate = [1−absorbance of the experimental group/control group absorbance]×100%.

### Cell apoptosis

Cells in the logarithmic growth phase were seeded in 6-well plates at a density of 10^5^/mL and incubated at 37°C in an atmosphere containing 5% CO_2_, 1% O_2_, and 94% N_2_, for 24 h. The cells were then harvested by trypsinization and washed once with phosphate-buffered saline (PBS, pH 7.4). After centrifugation, the cells were resuspended in 100 µL of binding buffer, and then stained with FITC-Annexin V and propidium iodide (Invitrogen Inc., Carlsbad, CA, USA) for analysis of cell apoptosis. The reaction was developed under dark conditions for 10 min, after which 400 µL of binding buffer was added to the wells. Cells were counted using a flow cytometer (Model BD LSR II; BD Bioscience, San Jose, CA, USA) (channel selection FL1 and FL2).

### Survivin and Ki67 mRNA levels by semi-quantitative RT-PCR

After treatment with paclitaxel and oxygen-CNTs for 48 h, cells were harvested. Total RNA was extracted using Trizol (Takara Bio, Otsu, Japan), and cDNA was produced by reverse transcription and amplification using the reverse transcription-polymerase chain reaction kit (RT-PCR, Takara Bio, Otsu, Japan). The primers were synthesized by Sangon Co. (Shanghai, China). The primers for GADPH were 5′-ACC ACA GTC CAT GCC ATC AC-3′ (sense) and 5′-CCA CCA CCC TGT TGC TGT A-3′ (antisense) (471 bp). The primers for Ki67 were 5′-ATC GTC CCA GGT GGA AGA GTT-3′ (sense) and 5′-ATA GTA ACC AGG CGT CTC GTG G-3′ (antisense) (126 bp). The primers for survivin were 5′-AGC CCT TTC TCA AGG ACC AC-3′ (sense) and 5′-GCA CTT TCT TCG CAG TTT CC-3′ (antisense) (363 bp). PCR conditions were: 30 cycles at 94°C for 30 s, 60°C for 30 s, and 72°C for 30 s. The products were detected using 1.5–2% agarose gel (Invitrogen Inc., Carlsbad, CA, USA) electrophoresis. Results were analyzed using the Bio-rad Gel DocTM XR+ imaging system (Bio-Rad Laboratories Inc., Hercules, CA, USA).

### Caspase, LC3 and Beclin-1 protein expression by western blot

Cells were washed and then suspended in 100 µL of lysate (sodium dodecyl sulfate+phenylmethyl sulfonylfluoride, SDS+PMSF). The total protein concentration was determined using the bicinchoninic acid (BCA) assay and a microplate reader at 562 nm. After an equal amount of protein was loaded in each lane, the proteins were separated using 10% SDS w/v polyacrylamide gel electrophoresis (SDS-PAGE) and then transferred onto a polyvinylidene difluoride (PVDF) membrane. After blocking the membrane with 5% (w/v) skim milk, target proteins were immunodetected using specific antibodies. All primary antibodies were used in a 1∶1000 dilution and incubated overnight at 4°C. After washing with Tris-buffered saline with Ttween-20 (TBST) three times for 20 min each, horseradish peroxidase (HRP)-conjugated antibody (Wuhan Boster Biological Engineering Co., Wuhan, China) was added as the secondary antibody. Positive bands were detected using ECL Plus Western Blotting Detection Regents. The results were scanned using the Bio-Rad Chemi Doc™ XRS+imaging system (Bio-Rad Laboratories Inc., Hercules, CA, USA). Caspase-3, caspase-8, and caspase-9 mouse monoclonal antibodies and p27 rabbit monoclonal antibodies were purchased from Santa Cruz (Santa Cruz, CA, USA). β-Actin, LC3, and Beclin-1 rabbit monoclonal antibodies were purchased from Cell Signaling Technology (Danvers, MA, USA).

### Chemotherapeutic effect of oxygen-CNTs in tumor-bearing animal models

Six-week-old female Balb/c nu/nu mice were obtained from the Shanghai Animal Center (Shanghai, China). The animals were housed in sterilized cages with filtered air, 12-h light-dark cycle, and had free access to sterile water and food. After 1 week of acclimatization, breast cancer cells were injected into the right flanks of each mouse (5×10^6^ cells, twice a week). After the tumors were allowed to develop for 2 weeks, the animals were randomly grouped into one of three groups (n = 6/group). The animals in the vehicle group were administered an intraperitoneal injection of 0.9% normal saline. The animals in the paclitaxel group were intraperitoneally administered 20 µg/kg body weight paclitaxel each week. Finally, the animals in the oxygen-CNT group were intraperitoneally administered 5 mg/kg body weight oxygen-CNTs each week local injection with lots of dot around the tumor. At the end of the experiment, the animals were euthanized with a CO_2_ overdose and decapitated. Tumor tissue from each animal was excised and then weighed after the excess normal tissue was trimmed off. The inhibition rate was calculated using the formula inhibition rate = [(Weight_control group_−Weight _experimental group_)/Weight_control group_]×100%.

### Statistical analysis

For the in vitro cell culture study, each experiment was repeated at least three times. Data are presented as mean ± standard deviation from multiple independent experiments. For the in vivo animal study, we obtained similar results from two independent experiments, and only one representative data set was used. Statistical significance was analyzed using one-way ANOVA and Dunnett's test. All analyses were performed using SPSS 18.0 (SPSS Inc., Chicago, IL, USA). A P-value<0.05 was considered statistically significant.

## Results

### Physical and chemical characteristics of oxygen-CNTs

**Figure 1:**
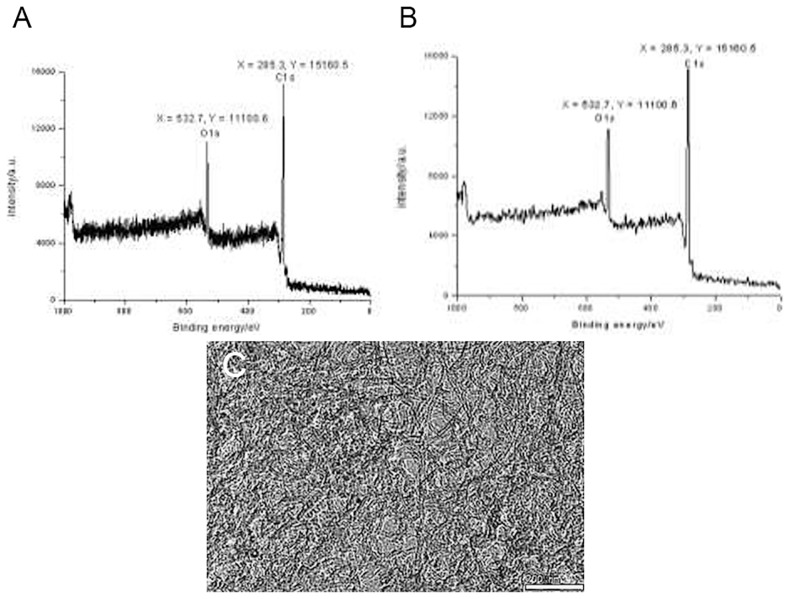
Physical and chemical characteristics of oxygen-CNTs. A. Analysis of the hydroxylation of carbon nanotubes by XPS. B. Data smoothing of the hydroxylation results. C. Transmission electronic microscopy image showing that the oxygen-CNTs well dispersed in solution.

Figure 1 indicates oxygen inclusion, with the oxygen absorption peak being between 600 eV and 500 eV, and the carbon absorption peak around 300 eV. These findings indicate that the CNTs contained oxygen (Fig. 1). The oxygen-CNTs contained 14.04% of oxygen, 82.68% of carbon, and 3.28% of nitrogen.

TEM pictures of functional oxygen single-walled carbon nanotubes after oxygen modification showed that the carbon nanotubes were evenly dispersed in solution (Fig. 1).

### Effect of oxygen-CNTs on the effects of paclitaxel

**Figure 2:**
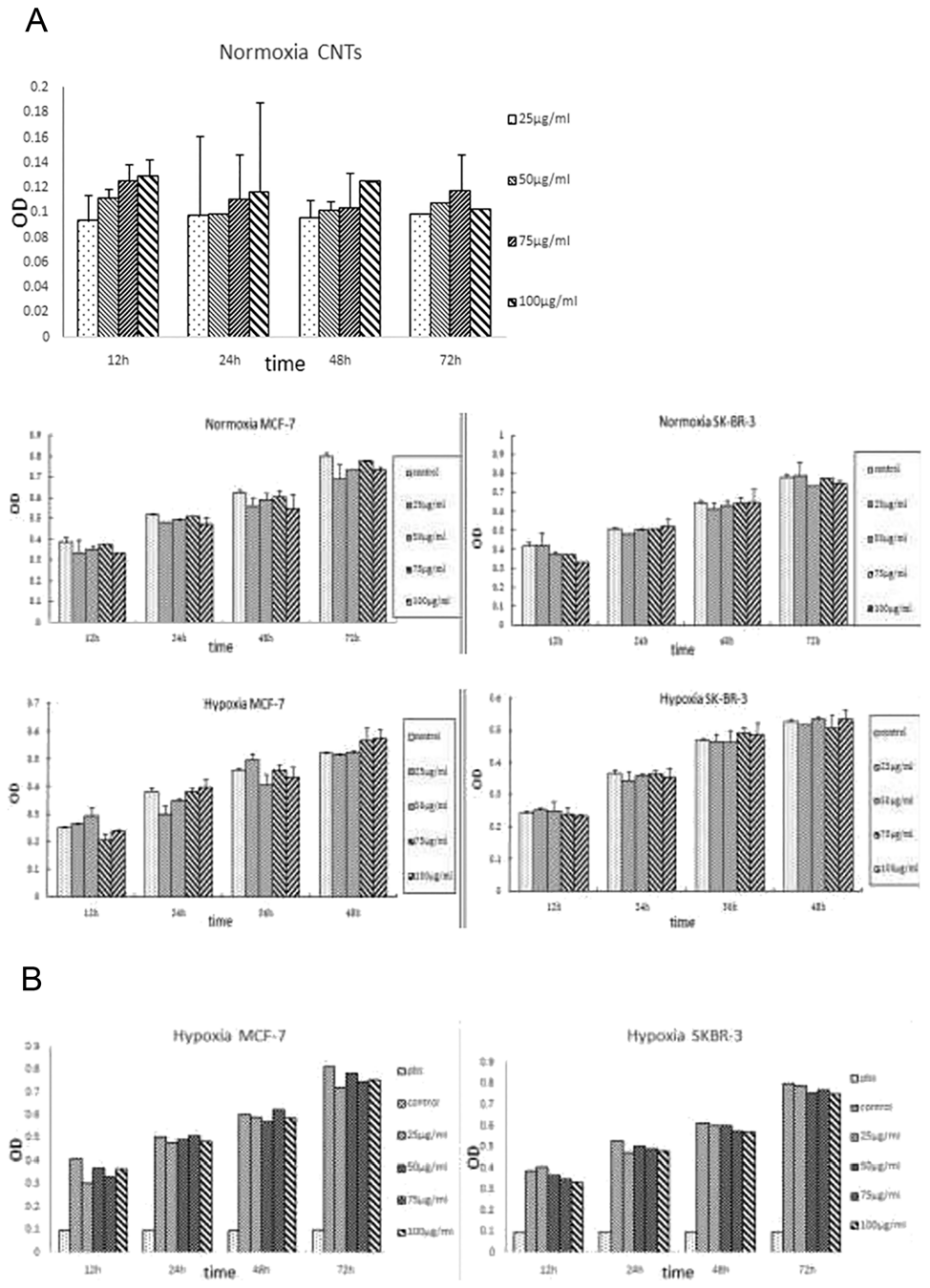
Effect of oxygen-CNTs on the inhibitory effect of paclitaxel. A. Effect of oxygen-CNTs on breast cancer cell activity under normoxic and hypoxic conditions. B. Impact of oxygen-CNTs combined with paclitaxel on cell activity under hypoxia.

The WST-1 assay showed that the oxygen-CNTs had no influence on the activity of MCF-7 cells in the normoxic environment (OD <0.2) (Fig. 2A). Moreover, under both normoxic and hypoxic conditions, the CNTs did not have a significant impact on cell proliferation and activity in both breast cancer cell lines. Gradient concentrations (25, 50, 75 and 100 µg/mL) of oxygen-CNTs with paclitaxel treatment showed increasing inhibition effects (Fig. 2B) on breast cancer cells compared with the control group. Cell activity of the paclitaxel group was reduced by 20% at 48 h, although the oxygen-CNTs+paclitaxel group showed a further decrease of 10–25%. Thus, these results demonstrate that oxygen-CNTs could improve the inhibitory effect of paclitaxel on cell proliferation.

**Figure 3:**
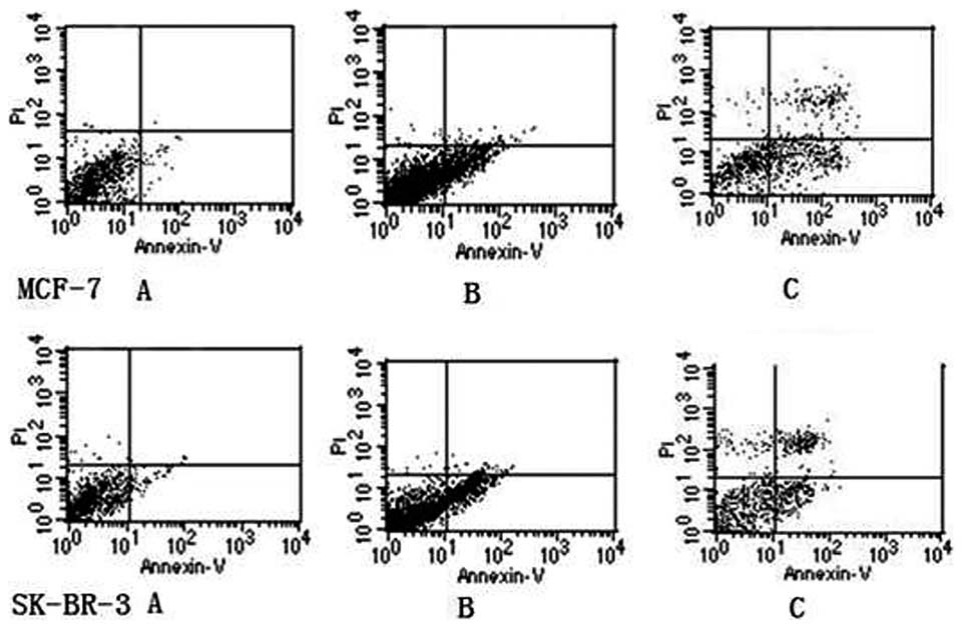
Effect of oxygen-CNTs and paclitaxel on cell apoptosis under hypoxia. A: hypoxia group; B: paclitaxel group; C: oxygen-CNT group.

The cell apoptosis rates determined using flow cytometry are shown in Figure 3. The apoptosis rate in the paclitaxel treatment group was 15% higher than that in the control group, and the apoptosis rate in the oxygen-CNT+paclitaxel group was 20% higher than that in the paclitaxel group (all P<0.05, Table 1), which indicated that oxygen-CNTs could enhance the effects of paclitaxel on the cell apoptosis rate in both cell lines.

**Table 1:** Effect of oxygen-CNTs and paclitaxel on cell apoptosis under hypoxia.

Group	MCF-7	SK-BR-3
Control	3.90%	7.30%
Paclitaxel	20.60%	24.70%
Paclitaxel+CNTs	44.50%	46.60%

### HIF-1α, survivin and Ki67 mRNA expression

**Figure 4:**
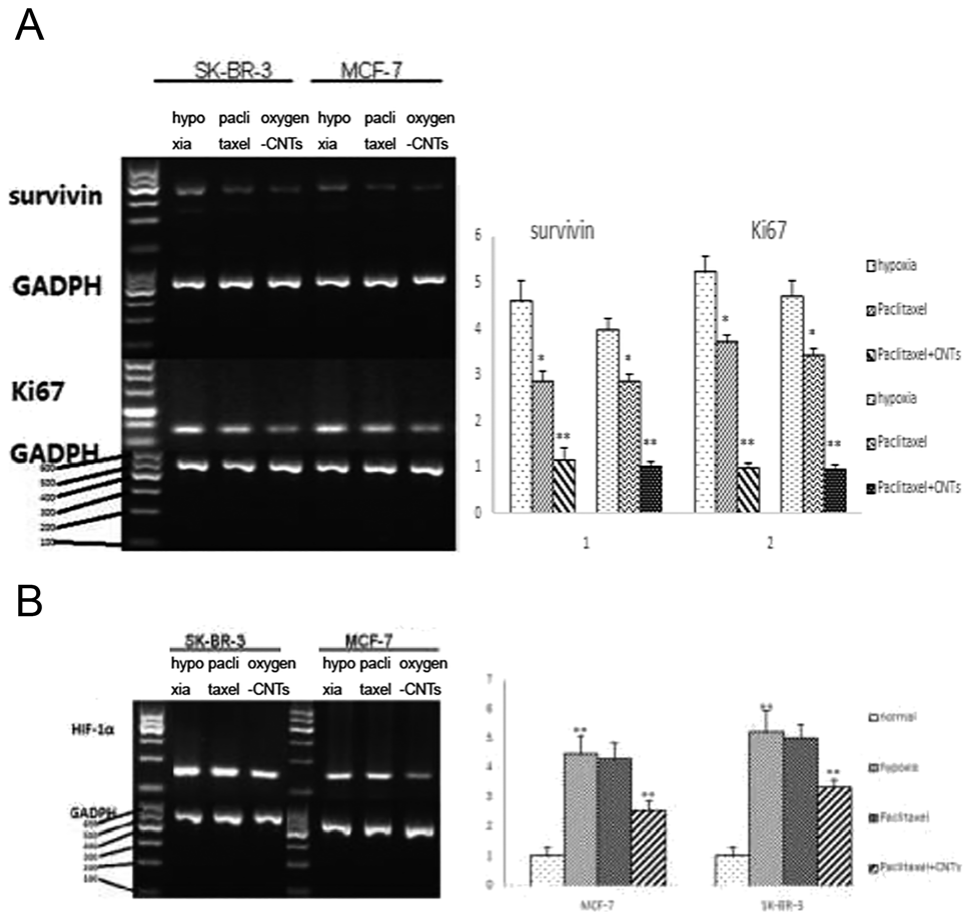
Effect of oxygen-CNTs and paclitaxel on survivin, Ki67 and HIF-1α mRNA expression. A. Oxygen-CNTs and affect survivin and Ki67 mRNA expression under hypoxic conditions. Typical figure (left) and analysis (right). 1: hypoxia group; 2: paclitaxel group; 3: oxygen-CNTs group. B. Effect of oxygen-CNTs on HIF-1α mRNA expression under normal and hypoxic conditions. Typical figure (left) and analysis (right). 1: normoxia group; 2: hypoxia group; 3: paclitaxel group; 4: oxygen-CNTs group.

RT-PCR showed that survivin and Ki67 mRNA expression were significantly reduced by 30–45% with the addition of oxygen-CNTs compared with paclitaxel only (P<0.05, Fig. 4). Under hypoxia, there was an apparent increase in HIF-1α mRNA expression compared with normal conditions, and the addition of oxygen-CNTs led to a reduction in HIF-1α expression (Fig. 4).

### Caspase, LC3 and Beclin-1 protein expression

**Figure 5:**
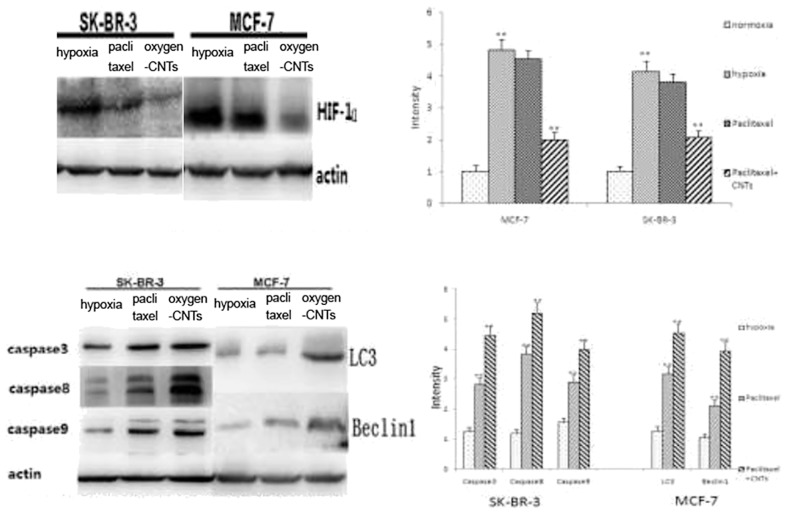
Effect of oxygen-CNTs and paclitaxel on the protein expression of HIF1α, caspase-3, caspase-8, caspase-9, LC3, and Beclin-1 under hypoxia. Typical figure (left) and analysis (right).

The expression of the apoptosis-related proteins caspase-3, caspase-8 and caspase-9, and of the autophagy-associated proteins LC3 and Beclin-1 was increased after 48 h of treatment, and the expression of these proteins further increased with the addition of oxygen-CNTs (all P<0.05, Fig. 5), indicating that CNTs significantly enhanced the apoptosis and autophagy effects of paclitaxel.

### Tumor weight estimation

No significant difference was observed in body weight between the experimental and vehicle groups. In the control group, tumor weight was modest, and partial rupture of the tumor capsule led to death in one case. Moreover, the oxygen-CNT+paclitaxel group showed a greater decline of 20–25% in tumor weight and volume compared with the paclitaxel group. Both experimental groups showed an obvious decline in tumor weight compared with the control group, and there was no ulceration or death in the experimental groups (P<0.05) (Table 2).

**Table 2:** Effect of oxygen-CNTs on chemotherapy in animal models of breast cancer.

Cells		Tumor weight (g)	Inhibition rate (%)
MCF-7	Control	2.008±0.141	
	Paclitaxel	0.968±0.105	51.79
	Paclitaxel+CNTs	0.548±0.074	72.71
SK-BR-3	Control	1.795±0.108	
	Paclitaxel	0.799±0.098	55.48
	Paclitaxel+CNTs	0.524±0.083	70.81

## Discussion

The aim of the present study was to assess the *in vivo* and *in vitro* effects of adding oxygen-CNTs to chemotherapy for hypoxic breast cancer. Results showed that in the normoxic and hypoxic environments, oxygen-CNTs had no significant effects on the growth of breast cancer cells. However, under hypoxia, oxygen-CNTs significantly enhanced the inhibitory effect of paclitaxel on cell proliferation, as well as the apoptosis rate. Under hypoxia, downregulation of HIF-1α and upregulation of caspase-3, caspase-8, caspase-9, LC3 and Beclin-1 were observed when paclitaxel was combined with oxygen-CNT. Furthermore, addition of oxygen-CNTs to chemotherapy was found to significantly reduce tumor weight in the tumor-bearing mice model.

CNTs present a high surface area, excellent chemical stability and are able to adsorb and conjugate with many chemical elements or macromolecules. Furthermore, they are able to enter into cells and to keep the bound molecules intact during transport [18], [19]. Therefore, CNTs are ideal agents for treatment delivery to tumor cells, and their first use was the delivery of chemotherapy directly into tumor cells [20]–[22]. In addition to drug delivery, CNTs have been used for antitumor immunotherapy, local antitumor hyperthermia therapy, for DNA delivery and for reactive oxygen species scavenging [23]. Furthermore, since CNTs do not rely upon active transport systems and are not subjected to multidrug resistance (MDR) mechanisms, they can be used to overcome MDR [24]. However, even if greatly promising, this approach does not overcome an important problem encountered in solid cancers: hypoxia.

Since tumors grow rapidly, the proliferation rate of tumor cells is higher than that of cells in the surrounding blood vessels, leading to severe local tissue hypoxia, which triggers the tumor cells into a series of stress responses [25], [26]. These responses lead to an increased resistance of cells to radiations and to chemotherapy [27], including paclitaxel [28], and leading to worst prognosis [29]. Therefore, delivering oxygen directly to the cells within solid tumors might be a way of overcoming hypoxia and hypoxia-induced resistance.

HIF-1 plays a key regulatory role in angiogenesis; it is associated with tumor development, invasion and metastasis, and affects the prognosis of most malignant tumors [30], [31]. The overexpression of HIF-1α has been detected in certain cancers such as gastric, breast and lung cancer [3], [32]. Moreover, the target genes of HIF-1α are involved in anaerobic glucose metabolism, oxygen transport, angiogenesis and vascular dilatation [32], which are key processes in tumorigenesis and malignant progression. It has also been reported that the expression of HIF-1α in most tumor tissues was related to tumor invasion, metastasis and prognosis [33]. HIF-1α is involved in the hypoxia-induced resistance to paclitaxel [34].

In the present study, under both normoxic and hypoxic conditions, oxygen-CNTs alone did not have an effect on the growth of breast cancer cells. However, oxygen-CNTs combined with paclitaxel showed more pronounced inhibition of the activity of breast cancer cells than paclitaxel alone under hypoxic conditions. These results are in agreement with previous studies that showed that the reoxygenation of hypoxic cancer cells led to better responses to paclitaxel treatment [35], [36]. Furthermore, oxygen-CNT treatment led to decreased HIF-1α levels, suggesting that delivery of oxygen to the cells decreased the expression of HIF-1α, decreasing the resistance to paclitaxel [34]. This resulted in the increase in proteins involved in apoptosis (caspase-3, caspase-8 and caspase-9) and autophagy (LC3 and Beclin-1), which are, at least in part, under HIF-1α control [37]–[39], as well as proteins that are involved in cell survival and growth (survivin and Ki67) [40], [41]. This resulted in a better in vivo tumor response to oxygen-CNTs and paclitaxel in the mice model. In addition, animals did not suffer from toxicity from the CNTs, as previously shown [42].

Therefore, it is possible that the addition of oxygen-CNTs under hypoxic conditions increased the availability of oxygen to the cells, increasing their sensitivity to paclitaxel and subsequently increasing apoptosis and autophagy. However, the specific mechanism by which oxygen-CNTs improve the chemotherapeutic effect of anti-cancer drugs needs to be further studied. The addition of oxygen to the cells did not seem to be toxic by itself. Therefore, we assume that the effects we observed were due to the chemo-sensitizing effects via decreased HIF-1α expression.

## Conclusions

Oxygen-CNTs improved the hypoxia status of breast cancer cells *in vitro* and *in vivo*, and significantly improved the chemotherapeutic effect of paclitaxel. Oxygen-CNTs may therefore be a potential chemotherapy sensitizer for breast cancer treatment.
